# Copper-anchored polysulfonamide-modified UiO-66-NH_2_/sodium alginate nanocatalyst for sustainable synthesis of 1,2,3-triazoles†

**DOI:** 10.1039/d4na01055h

**Published:** 2025-02-04

**Authors:** Samaneh Koosha, Ramin Ghorbani-Vaghei, Sedigheh Alavinia

**Affiliations:** a Department of Organic Chemistry, Faculty of Chemistry and Petroleum Sciences, Bu-Ali Sina University Hamadan 6517838683 Iran rgvaghei@yahoo.com ghorbani@basu.ac.ir; b Department of Organic Chemistry, Faculty of Chemistry, University of Guilan Rasht Iran

## Abstract

An effective nanocomposite comprising a metal–organic framework and porous polysulfonamide-sodium alginate (SA-PS) was developed for phenyl triazole production. The Cu(i) ions were uniformly distributed on the as-prepared UiO-66-NH_2_@SA-PS matrix, coordinated by sulfonamide groups in a bidentate bridging pattern (UiO-66-NH_2_@SA-PS/CuI). The nanocatalyst UiO-66-NH_2_@SA-PS/CuI demonstrated exceptional performance in the synthesis of 1,2,3-triazole derivatives, facilitating high product yields in the reaction of various aryl boronic acids, phenylacetylene, and sodium azide under mild conditions.

## Abstract 2

An effective nanocomposite comprising a metal–organic framework and porous polysulfonamide-sodium alginate (SA-PS) was developed for phenyl triazole production.

## Graphical abstract

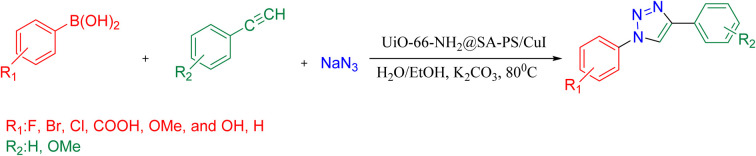

## 1. Introduction

**Scheme 1:**
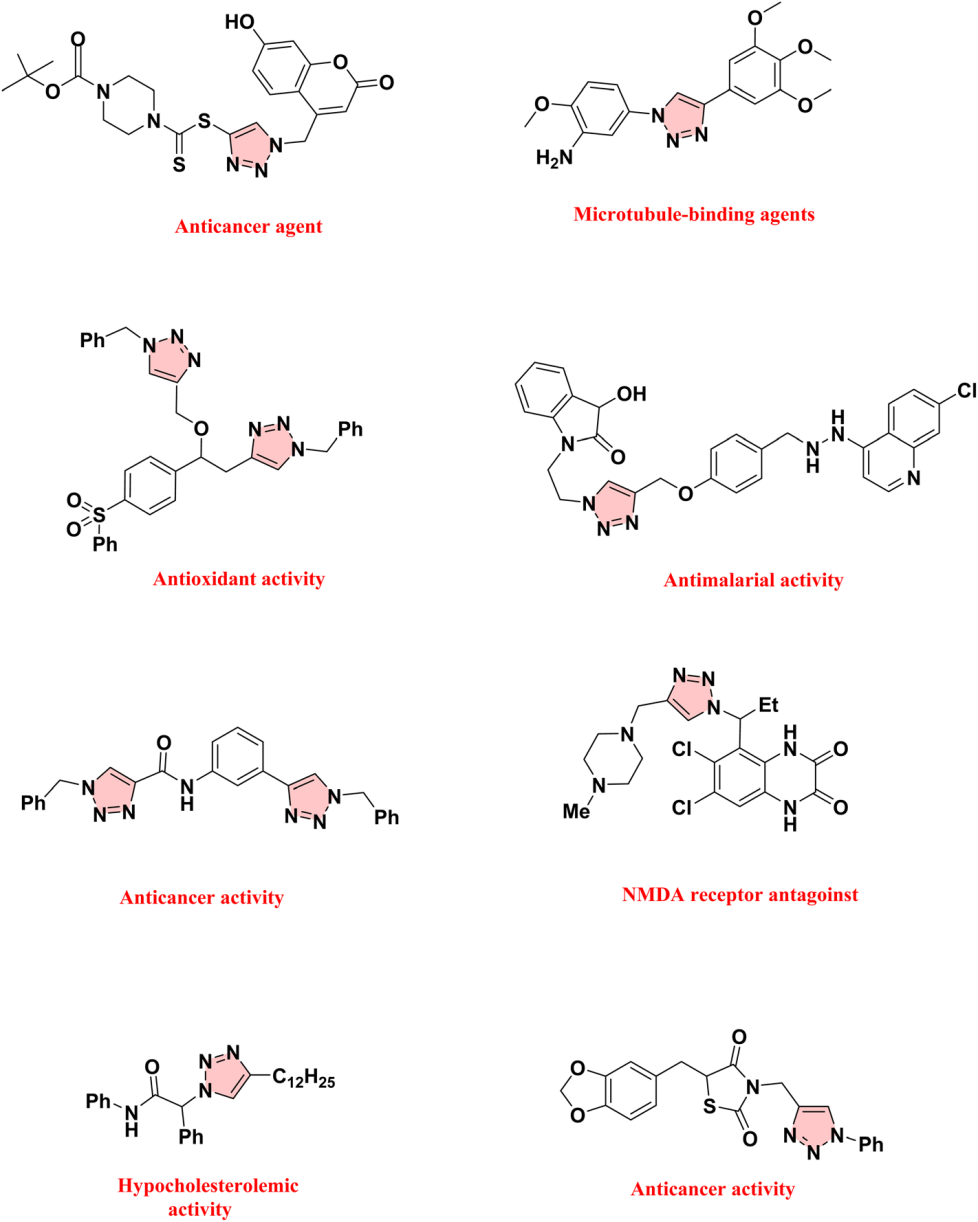
Triazole derivatives with biological activity.

The 1,2,3-triazoles are versatile compounds with significant biological and industrial importance. Due to their many potential structural and pharmacological characteristics, they have become a preferred scaffold in medicinal chemistry.^1^ Their synthesis, primarily through Huisgen cycloaddition, provides a robust method for accessing a variety of derivatives, thereby supporting extensive research and development in multiple fields of chemistry and medicine.^2^ Click chemistry utilizes small fundamental molecular units compiled in combinatorial libraries. These structures rely on selective, straightforward carbon–heteroatom bonds (C–X–C), allowing them to recombine in one-step reactions.^3^ This approach aligns with green chemistry principles by enabling the rapid creation of a wide variety of compounds. Click chemistry is currently more prevalent in biological contexts than it is in the chemical phase of drug development, which offers an opportunity to enhance the reactions (Scheme 1).^4^ Recent reviews have comprehensively examined the progress of CuAAC reactions, and numerous catalytic copper systems have been reported.^5–8^ These systems are categorized into Cu(i) sources, Cu(ii) salts or complexes, Cu nanoparticles, Cu MOFs^6^ and others. Typical catalysts include Cu(i) sources such as CuI, CuBr, and CuCl.^5–9^ Homogeneous Cu(ii) salts such as CuSO_4_, CuCl_2_ and Cu(OAc)_2_, in combination with sodium ascorbate, have been shown to be effective catalysts for CuAAC reactions.^10^ Therefore, a variety of polymer-supported copper catalysts, such as mPAN-Cu(ii),^11^ Cu(i)/PVPP-Fe_3_O_4_,^12^ Cu@CB-n,^13^ Cu(i)NVPMBA,^14^ PS-PEG-TD2-CuSO_4_,^15^ PANFPABuBuX@CuX,^16^ and CuX-PBPTP^17^ have been successfully employed for this reaction.

The development of biodegradable solid catalysts utilizing renewable biopolymers presents a feasible approach to achieving safer and more sustainable organic synthesis methods, aligning with the principles of green chemistry.^18^ In recent decades, heterogeneous catalytic processes have garnered significant interest for their potential to improve the sustainability of chemical reactions.^19,20^ By leveraging renewable biopolymers, these catalysts provide an environmentally friendly alternative to conventional systems, enhancing both the safety and efficiency of organic synthesis.^21^

Sodium alginate is a water-soluble biopolymer commonly found in aquatic phytoplankton. This biodegradable polymer consists of chains comprising two monomeric components: 1,4-linked β-d-mannuronic acid and α-l-guluronic acid. Due to the presence of hydroxy (–OH) and carboxylate (–COO^−^) functional groups along its chain, alginate exhibits electrostatic interactions.^22^ However, similar to other polymeric gels, pure ionically cross-linked alginate hydrogels possess certain disadvantages, such as structural instability and low mechanical strength, which can limit their practical applications. To improve the physical properties of sodium alginate, graft copolymerization and nanocasting techniques are practical and efficient methods.^23^

MOFs are highly promising materials due to the versatility and tunability of their orderly crystalline structures.^24,25^ Through the integration with carefully engineered polymers to form MOF composites, the shortcomings of pure MOFs can be compensated for, thus achieving synergistic effects for the enhancement and expansion of their performance. MOF/polymer nanocomposites have emerged as promising materials for various applications, including organic reactions.^26–28^

The combination of sodium alginate, polysulfonamide and the metal–organic framework provides an amphiphilic environment for the reaction catalyst. The prepared substrate facilitates effective loading of copper iodide by forming strong bonds with copper iodide nanoparticles. Copper iodide nanoparticles also play a crucial role in improving the reaction conditions as active catalytic species.

In this study, a novel catalyst was designed using the readily accessible UiO-66-NH_2_-functionalized porous sodium alginate-polysulfonamide as a multifunctional heterogeneous support for the immobilization of CuI NPs. The MOF@polymer core–shell catalyst offers a tailored and selective environment for the synthesis of 1,2,3-triazole compounds from the reaction of aryl boronic acids, phenylacetylene, and sodium azide through Huisgen cycloaddition. This study reports the 1,2,3-triazole synthesis, emphasizing the use of a porous sodium alginate-polysulfonamide/copper nanocatalyst supported by a metal–organic framework. This method synthesizes compounds with high efficiency in the presence of an environmentally friendly catalyst (Scheme 1).

## 2. Experimental section

### 2.1 UiO-66-NH_2_ synthesis

UiO-66-NH_2_ was synthesized using a solvothermal method. Initially, a mixture of 2-aminoterephthalic acid (0.12 g) and zirconium chloride (0.14 g) was dispersed in DMF solvent (50 mL). Subsequently, the solution was transferred to a Teflon-lined autoclave and maintained at 130 °C for 24 h. The sample was immersed in 10 mL of hot EtOH and 10 mL of DMF for 24 h to remove any residual acids trapped inside the pores. Subsequently, it was filtered and dried at 100 °C overnight to obtain the UiO-66-NH_2_.^29,30^

### 2.2 Porous SA-PS synthesis

For polymerization, sodium alginate (0.25 g), *p*-styrene sulfonamide (1.00 g), and SiO_2_ nanoparticles (0.05 g) were stirred with a magnet in distilled water (10 mL) at 70 °C. After 10 min, 0.04 g of ammonium persulfate was added to the mixture, and the reaction mixture was stirred for 2 h with a magnetic stirrer until SiO_2_/SA-PS nanoparticles were synthesized and washed with H_2_O (5 mL) and EtOH (5 mL) to obtain a solid material, which was dried at room temperature under vacuum conditions. Then, the silica in the SiO_2_/SA-PS was selectively removed with HF solution. HF solution (10 mL), deionized water (10 mL), and SiO_2_/SA-PS nanoparticles (0.5 g) were added in a container. Then, the mixture was stirred at room temperature for 6 h to obtain the prepared porous polymer, washed with H_2_O (50 mL) and dried at 50 °C. ^31^

### 2.3 Preparation of UiO-66-NH_2_@SA-PS nanocomposite

The new nanocomposite UiO-66-NH_2_@SA-PS was synthesized by the reaction of UiO-66-NH_2_ with porous SA-PS. UiO-66-NH_2_ (0.15 g) was dispersed in DMF (5 mL). Then, porous SA-PS (0.12 g) in 5 mL DMF was added dropwise to the mixture and stirred for 24 h at 50 °C with a magnetic stirrer. Finally, the sediment obtained was dried in an oven at 110 °C for 24 h to remove the residual solvent to gain UiO-66-NH_2_@SA-PS.^31^

### 2.4 Preparation of UiO-66-NH_2_@SA-PS/CuI nanocomposite

**Scheme 2:**
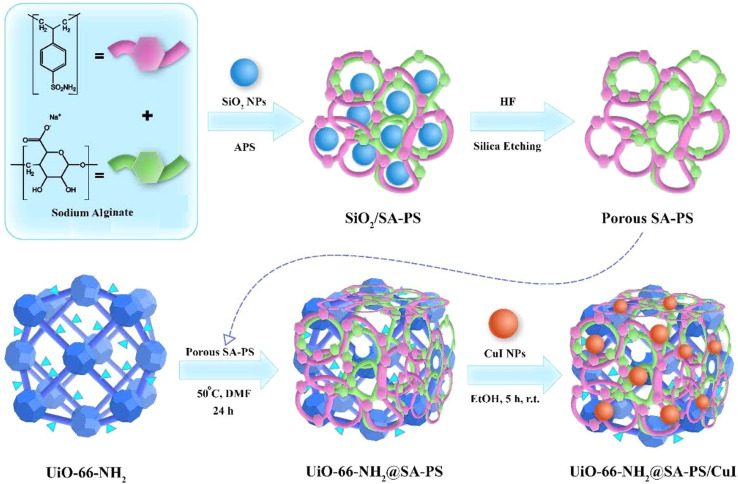
Schematic pathway for the fabrication of UiO-66-NH_2_@SA-PS/CuI.^31^

Subsequently, the resulting UiO-66-NH_2_@SA-PS powder (0.1 g) and CuI nanoparticles (0.05 g) were added to 20 mL acetonitrile, and the mixture was stirred at 50 °C with a magnetic stir bar for 12 h. The resulting powder was collected by filtration and washed thoroughly with deionized water (20 mL) and ethanol (20 mL) to remove the unreacted reactant, and then dried (Scheme 2).^31^

### 2.5 General procedure for 1,2,3-triazole synthesis

**Scheme 3:**
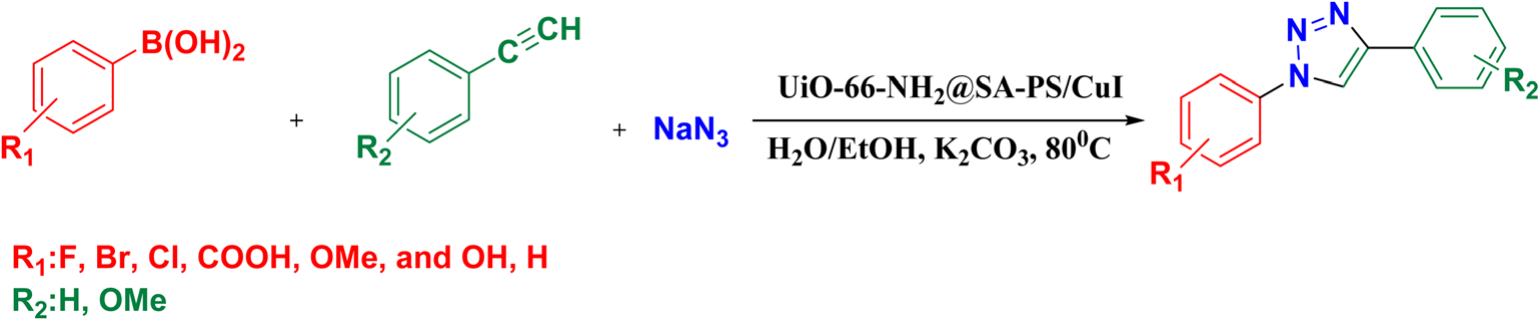
Schematic pathway for the fabrication of 1,2,3-triazole using UiO-66-NH_2_@SA-PS/CuI nanocomposite.

A mixture of potassium carbonate (138 mg, 1 mmol), phenylboronic acid (122 mg, 1 mmol), sodium azide (195 mg, 3 mmol) and UiO-66@SA/PSA@CuI (10 mg, 0.64 mol%) in H_2_O/EtOH (2 mL) was stirred at 80 °C for 1 h. Then, phenylacetylene (102 mg, 1 mmol) was added to the mixture and stirred for an appropriate time. The progress of reaction was checked using TLC (*n*-hexane/ethyl acetate = 10 : 3). After completion of the reaction, the mixture was cooled and then centrifuged to separate the catalyst. After drying, the sediment from the reaction was extracted (ethyl acetate/water = 1 : 2, 30 mL), dried and evaporated, and finally, the crude product was washed with hot *n*-hexane (Scheme 3). The amount of copper incorporated in the support was 4.064%, as corroborated using inductively coupled plasma optical emission spectroscopy (ICP-OES).

## 3. Result and discussion

### 3.1 Catalyst activity

Optimization reactions were performed for the reaction of phenylboronic acid 1a, sodium azide 2a, and phenylacetylene 3a to determine the best reaction conditions to yield 1,2,3-triazoles (Table 1) in K_2_CO_3_. UiO-66-NH_2_@SA-PS/CuI is essential for the abovementioned reaction. The best result was achieved when employing 10 mg of UiO-66-NH_2_@SA-PS/CuI catalyst (entry 3), which produced yields of 98%. Further increasing the catalyst dosage beyond 10 mg did not significantly elevate the product yields (entry 4). The reaction did not proceed in the absence of K_2_CO_3_ (entry 5). In addition, we performed experiments to investigate different bases for the model reaction. Notably, K_2_CO_3_ emerged as the most advantageous base, yielding both the highest output and the shortest reaction time when the model reaction was conducted. This highlights the critical role of base selection in optimizing reaction conditions for the click reaction (entries 6–8).

**Table 1:** Model reaction optimization^a^

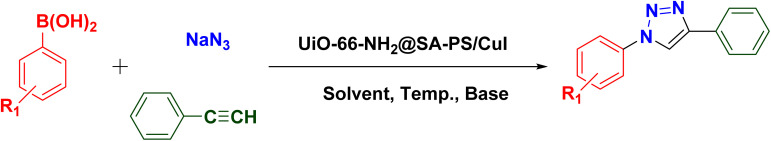				
Entry	Cat. (mol%)	Base (mmol)	Solvent	Yield^b^ (%)
1	—	K_2_CO_3_ (1)	H_2_O/EtOH	N.R.
2	0.32	K_2_CO_3_ (1)	H_2_O/EtOH	85
3	0.64	K_2_CO_3_ (1)	H_2_O/EtOH	98
4	0.96	K_2_CO_3_ (1)	H_2_O/EtOH	98
5	0.64	—	H_2_O/EtOH	55
6	0.64	KOH (1)	H_2_O/EtOH	42
7	0.64	Na_2_CO_3_ (1)	H_2_O/EtOH	39
8	0.64	Cs_2_CO_3_ (1)	H_2_O/EtOH	27
9	0.64	K_2_CO_3_ (1)	PEG	30
10	0.64	K_2_CO_3_ (1)	H_2_O	10
11	0.64	K_2_CO_3_ (1)	EtOH	40
12	0.64	K_2_CO_3_ (1)	DMF/H_2_O	59
13	0.64	K_2_CO_3_ (1)	CH_3_CN/H_2_O	68
14	(0.64	K_2_CO_3_ (1)	PEG/H_2_O	72
15	0.64	K_2_CO_3_ (1)	H_2_O/EtOH	80^c^
16	0.64	K_2_CO_3_ (2)	H_2_O/EtOH	84
17	0.64	K_2_CO_3_ (0.5)	H_2_O/EtOH	60

a Reaction conditions: phenylboronic acid (1 mmol), NaN_3_ (3 mmol), phenylacetylene (1 mmol), K_2_CO_3_ (1 mmol), and UiO-66-NH_2_@SA-PS/CuI (0.64 mol%) in H_2_O/EtOH (1 : 1, 2 mL) for 90 min.

b Isolated yield.

c The reaction was performed at 80 °C.

The choice of solvent significantly influenced the effectiveness of the click reaction, as evidenced by the varying yields observed across different solvents, which are summarized in Table 1. The catalytic potential in a model reaction was studied using various organic solvents, including PEG, DMF/H_2_O, CH_3_CN/H_2_O, PEG/H_2_O, H_2_O/EtOH, ethanol and water (entries 9–15). When only PEG was employed, its high viscosity resulted in low solubility for the synthesized catalyst, which consequently led to a diminished yield (entry 9). In contrast, using only water allowed phenylboronic acid and the base to dissolve effectively; however, phenylacetylene remained insoluble, resulting in poor yields (entry 10). Conversely, while phenylacetylene dissolved well in ethanol (EtOH), the other components did not, leading to similarly low yields (entry 11). The results presented in Table 1 demonstrate that binary solvent systems are significantly more effective, as they enable the dissolution of all catalytic components (entries 12–14). The EtOH/H_2_O mixture was ultimately selected due to its high catalytic activity and the fact that both solvents are environmentally friendly (entry 2). The evaluation of temperature indicated that 80 °C provides the highest yield (entries 15 *vs.* 2). Ultimately, our results revealed that the optimal dosage of K_2_CO_3_ catalyst for this reaction was 1 mol (entries 16–17).

Furthermore, the efficiency of the UiO-66-NH_2_@SA-PS/CuI catalyst was compared as shown in Table 2, entries 1–6. The results indicate that the high performance of the synthesized catalyst is related to synergistic effects of UiO-66-NH_2_, porous SA-PS, and CuI NPs (entry 6 *vs.* 1–5).

**Table 2:** Comparison efficiency of UiO-66-NH_2_@SA-PS/CuI with different catalysts

Entry	Catalyst	Base (mmol)	Yield (%)
1	UiO-66-NH_2_ (10 mg)	K_2_CO_3_	40
2	UiO-66-NH_2_@SA (10 mg)	K_2_CO_3_	48
3	UiO-66-NH_2_@ SA-PS (10 mg)	K_2_CO_3_	50
4	CuI (10 mg)	K_2_CO_3_	60
5	UiO-66-NH_2_@CuI (10 mg)	K_2_CO_3_	70
6	UiO-66-NH_2_@SA-PS/CuI (0.64 mol%)	K_2_CO_3_	98

To investigate the generality of the copper-catalyzed alkyne–azide cycloaddition reaction, the scope and limitations of this method were investigated using 10 diverse phenylboronic acids. The one-pot reaction of different substituted aryl boronic acids in the presence of H_2_O/EtOH and K_2_CO_3_ in MeOH using UiO-66-NH_2_@SA-PS/CuI at 80 °C gave 1,2,3-triazoles. In this catalytic system, the aryl boronic acids bearing either electron-donating or electron-withdrawing groups on the aromatic ring (F, Br, Cl, COOH, OMe, and OH) readily undergo the reaction to afford the corresponding products in good to excellent yields. As shown in Table 3, the presence of an electron-withdrawing substitute in phenylboronic acid extended the reaction time. A simple process to purify the products, without chromatographic purification, was achieved in this study.

**Table 3:** UiO-66-NH_2_@SA-PS/CuI-catalyzed CuAAC reactions based on the various boronic acids and alkynes^a^

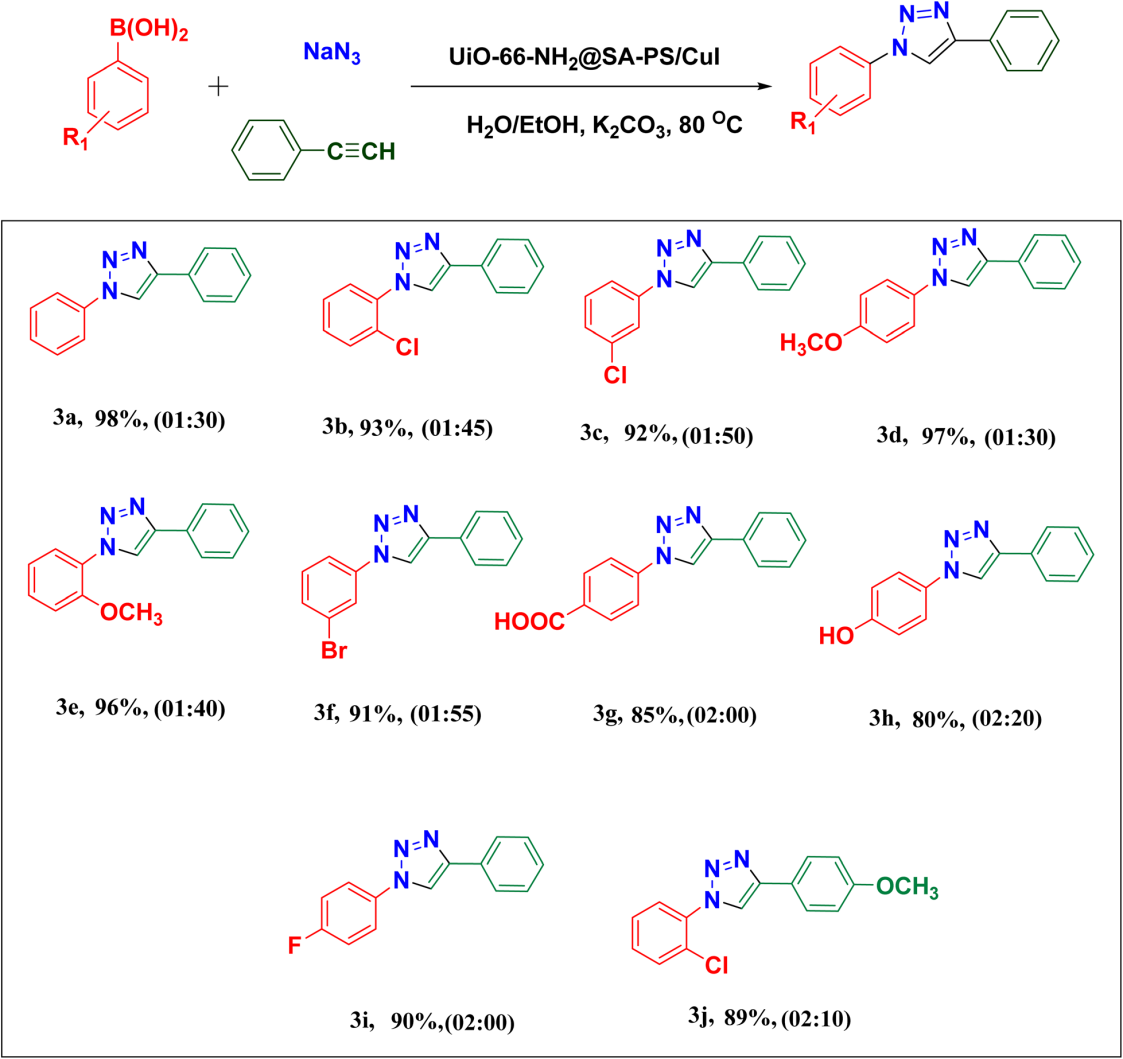

a Reaction conditions: phenylboronic acid (1 mmol), NaN_3_ (3 mmol), phenylacetylene (1 mmol), K_2_CO_3_ (1 mmol) and UiO-66-NH_2_@SA-PS/CuI (0.64 mol%) in H_2_O/EtOH (1 : 1, 2 mL). Isolated yield.

**Scheme 4:**
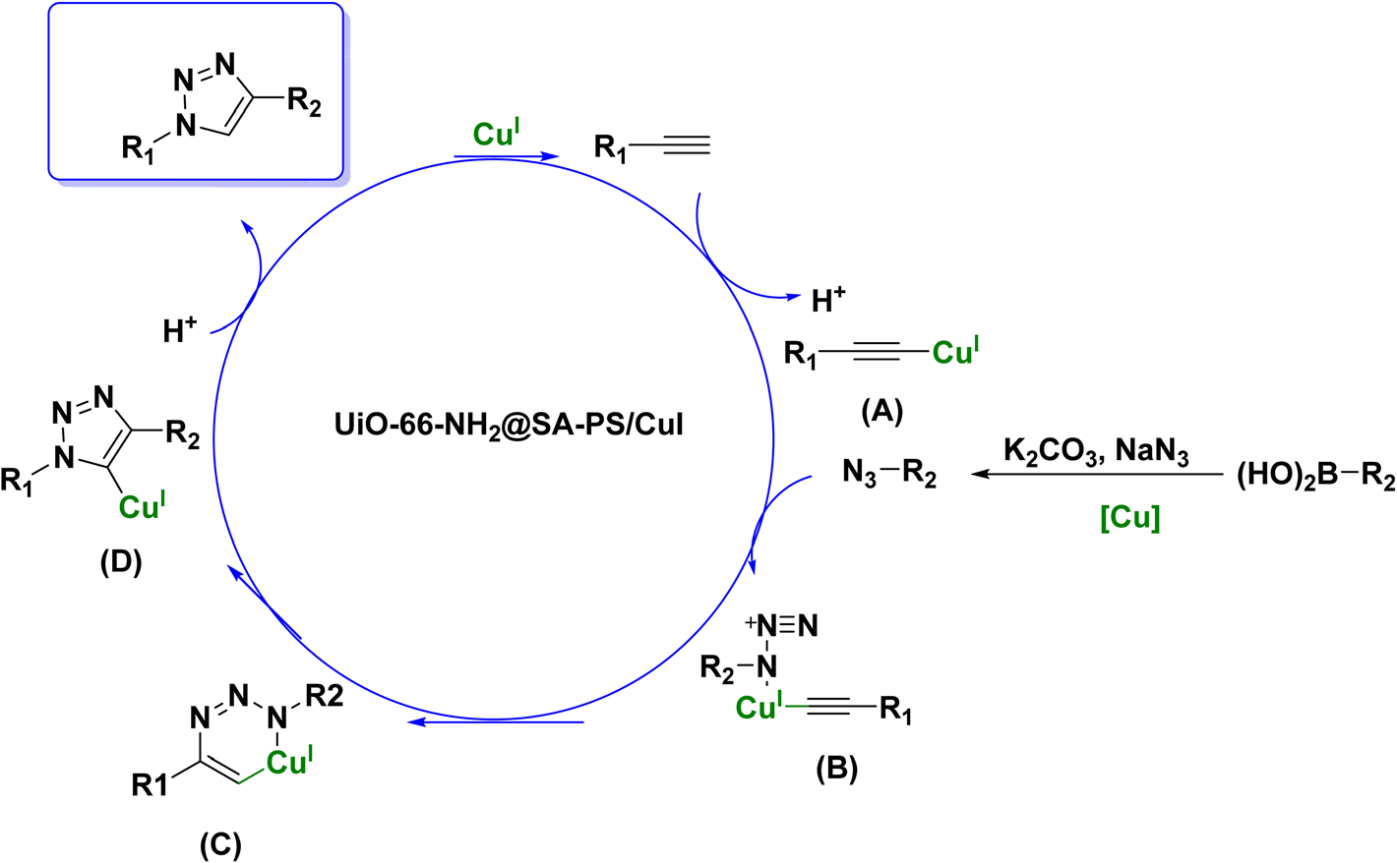
Proposed mechanism for the UiO-66-NH_2_@SA-PS/CuI-catalyzed CuAAC reaction.

Based on literature reports,^32^ a plausible mechanism for one-pot CuAAC synthesis has been proposed (Scheme 3), wherein NaN_3_ plays the role of azide precursor. Cu(i) species are prone to oxidation to Cu(ii) due to their thermodynamic instability.^9^ The reaction begins with a boronic acid derivative reacting with sodium azide (NaN_3_) to create an azide group. The UiO-66-NH_2_@SA-PS/CuI catalyst then coordinates with the terminal alkyne, forming a copper–acetylide complex A and releasing a proton (intermediate A). Subsequently, the azide group coordinates with the copper–acetylide complex, leading to a cycloaddition reaction between the azide and the alkyne to produce a triazole ring (intermediates B and C). Thirdly, rearrangement of intermediate C leads to the formation of the copper-metalated triazolide D and releases the catalytic Cu(i) species. Finally, the intermediate C captures a proton to produce the target triazole and regenerate the Cu(i) intermediate. In addition, UiO-66-NH_2_@SA-PS in this catalytic cycle serves as an efficient ligand to tune the activity of Cu(i) species on the catalyst (Scheme 4).^33^

**Fig. 1:**
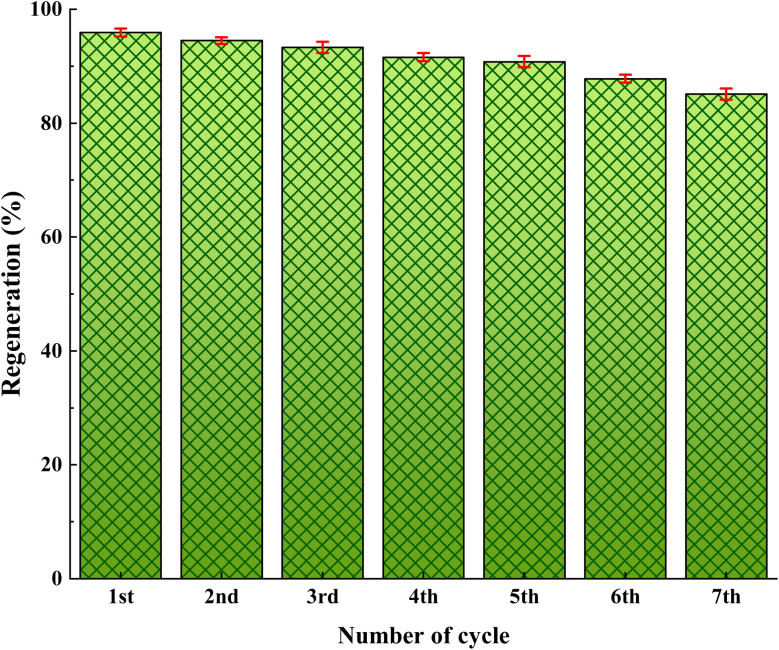
Recyclability performance of UiO-66-NH_2_@SA-PS/CuI.

Finally, for practical applications, the recycling of the catalyst was investigated using the model reaction. After completion of the reaction, the catalyst was conveniently and efficiently recovered from the reaction mixture by suction and then subjected to the next run directly without further treatment. As shown in Fig. 1, the catalyst can be reused consecutively at least seven times with comparatively little loss of its catalytic activity. The ICP-AES analysis showed that Cu content in the catalyst after the first run is 3.88%, which is slightly lower than the 4.064% Cu content in fresh catalyst. The slight decrease of the catalytic activity during recycling may be owing to the leaching of copper from the catalyst.

The findings from the click reaction conducted under different conditions are presented in Table 4. This investigation proposes UiO-66-NH_2_@SA-PS/CuI as an environmentally favorable nanocatalyst, leading to improved reaction conditions and enhanced performance (entry 7). The synergy of UiO-66-NH_2_, porous SA-PS and copper iodide nanoparticles shows higher efficiencies and superior catalyst utilization. The UiO-66-NH_2_@SA-PS/CuI catalyst exhibits high yields in short reaction times at mild temperature, with high loading of the catalyst, making performing the reaction effective with only a small amount of the catalyst. Moreover, the UiO-66-NH_2_@SA-PS/CuI catalyst is partly formed by sodium alginate and polysulfonamide as a non-toxic biopolymer, which makes the catalyst more biodegradable and environmentally friendly. Additionally, the UiO-66-NH_2_@SA-PS/CuI catalyst can be separated from the reaction mixture by centrifugation and easily reused seven times. This suggests that the current method may be a more ideal choice for achieving optimal results in the click reaction.

**Table 4:** Comparison studies

Entry	Catalyst	Base	Solvent	*T* (^o^C)	Time (h)	Yield (%)	Ref.
1	Cu(C_4_H_3_N(CHNCH_3_))_2_	—	EtOH/H_2_O	30	11.5	96	34
2	Cu–NHC@SiO_2_	Sodium ascorbate	Ethylene glycol	60	1	96	35
3	Copper diacetate	—	H_2_O	20	1	94	36
4	CuO–NiO	—	H_2_O	60	3	85	37
5	Cu/PANI		Ethylene glycol	20	3	75	38
6	Cu/Al_2_O_3_	K_2_CO_3_	Ball-milling condition		2	91	39
**7**	**UiO-66-NH**_**2**_ **@SA-PS/CuI**	**K**_**2**_ **CO**_**3**_	**H**_**2**_ **O/EtOH**	**80**	**0.64**	**98**	**This Work**

## 4. Conclusion

An efficient and environmentally friendly method was introduced for 1,4-disubstituted-1,2,3-triazole synthesis using utilizing UiO-66-NH_2_@SA-PS/CuI through click reactions. The easy process avoids the use of hazardous azides, with simple purification. It could be studied for other reactions to be applied for high-yield processes. The excellent activity of the catalyst has been ascribed to the coordination of sulfonamide groups with copper species. Moreover, there is no significant loss of catalytic activity when the catalyst is reused up to seven runs. Therefore, the high catalytic performance, reusability of catalyst, wide substrate scope, short reaction time, and mild conditions are the salient features of this green catalytic process, which make it more competitive for practical applications.

## Data availability

Data is provided within the manuscript or ESI.†

## Conflicts of interest

There are no conflicts to declare.

## Supplementary Material

NA-007-D4NA01055H-s001
