# Supplementary material for: Copper-anchored polysulfonamide-modified UiO-66-NH2/sodium alginate nanocatalyst for sustainable synthesis of 1,2,3-triazoles
Source: Nanoscale Adv. 2025 Feb 4;7(7):1937–45. doi: 10.1039/d4na01055h (PMC11808793; doi:10.1039/d4na01055h)
Supplement: NA-007-D4NA01055H-s001 [file NA-007-D4NA01055H-s001.pdf]

Supplementary Information (SI) for Nanoscale Advances.
This journal is © The Royal Society of Chemistry 2025

Supporting information

Copper-Anchored Polysulfonamide-Modified UiO-66-NH_2/Sodium Alginate Nanocatalyst for Sustainable Synthesis of 1,2,3-Triazoles

Samaneh Koosha[a], Ramin Ghorbani-Vaghei*[,a,b], Sedigheh Alavinia[a]

[a]*Department of Organic Chemistry, Faculty of Chemistry and Petroleum Sciences, Bu-Ali Sina University, 6517838683, Hamadan, Iran. E-mail: rgvaghei@yahoo.com; ghorbani@basu.ac.ir.*

[b]*Department of Organic Chemistry, Faculty of Chemistry, University of Guilan, Rasht, Iran*

Spectral data for the compounds

^{1}H NMR (400 MHz, DMSO) δ 9.10 (s, 1H), 7.92 (t, *J* = 10.1 Hz, 1H), 7.80 (d, *J* = 7.7 Hz, 1H), 7.78 – 7.68 (m, 1H), 7.47 (d, *J* = 8.4 Hz, 1H), 7.32 (d, *J* = 4.5 Hz, 2H), 7.14 (d, *J* = 7.4 Hz, 1H), 7.04 – 6.93 (m, 2H). ^{13}C NMR (101 MHz, DMSO) δ 148.20, 134.53, 134.29, 132.32, 130.58, 129.93, 129.50, 128.90, 128.79, 128.45, 126.72, 126.47, 125.82, 125.26. IR (KBr) 3390.31, 3356.85, 3285.12, 2125.74, 2043.25, 1451.38, 1005.69. 639.43 cm^{-1}.

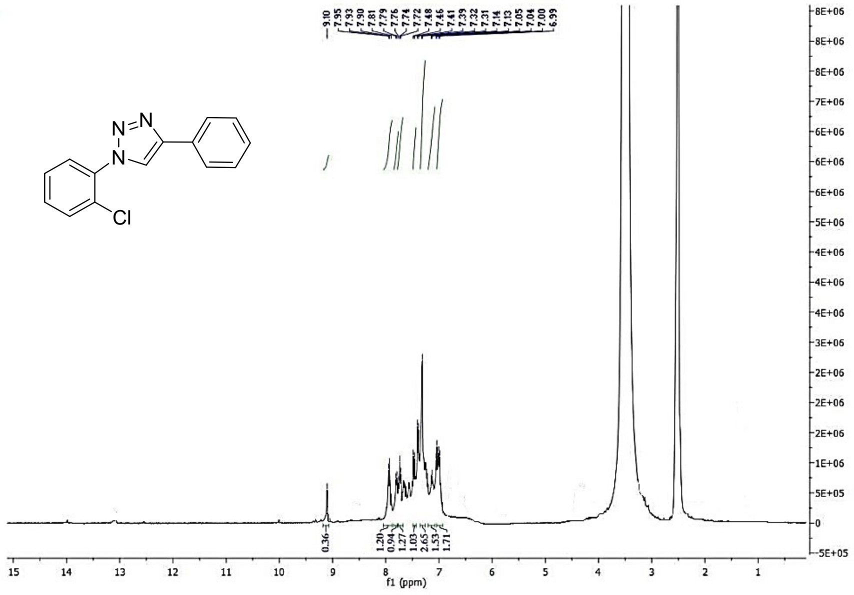

Figure S1. H NMR of 2-(4-Phenyl-1H-1,2,3-triazol-yl)chlorobenzene

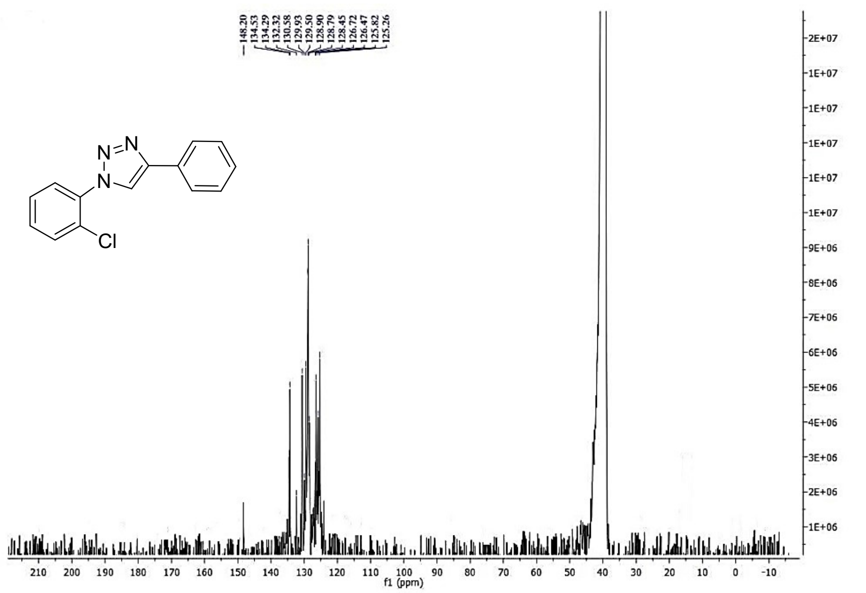

Figure S2. ^{13}C NMR of 2-(4-Phenyl-1H-1,2,3-triazol-yl)chlorobenzene

^{1}H NMR (400 MHz, DMSO) δ 8.92 (s, 1H), 7.88 (dd, *J* = 38.6, 6.3 Hz, 2H), 7.83 (d, *J* = 5.7 Hz, 1H), 7.64 (d, *J* = 7.6 Hz, 1H), 7.55 (t, *J* = 7.7 Hz, 1H), 7.47 (t, *J* = 7.0 Hz, 1H), 7.35 (t, *J* = 7.1 Hz, 2H), 7.16 (t, *J* = 7.5 Hz, 1H), 3.86 (s, 3H). ^{13}C NMR (101 MHz, DMSO) δ 165.45, 146.67, 131.98, 131.44, 130.74, 129.43, 128.54, 128.09, 126.38, 125.80, 123.92, 122.35, 121.36, 113.52, 56.62. IR (KBr) 3391.5, 3357.02, 2146.14, 2023.29, 1586.4, 1474.13, 1130.7, 1002.67 cm^{-1}.

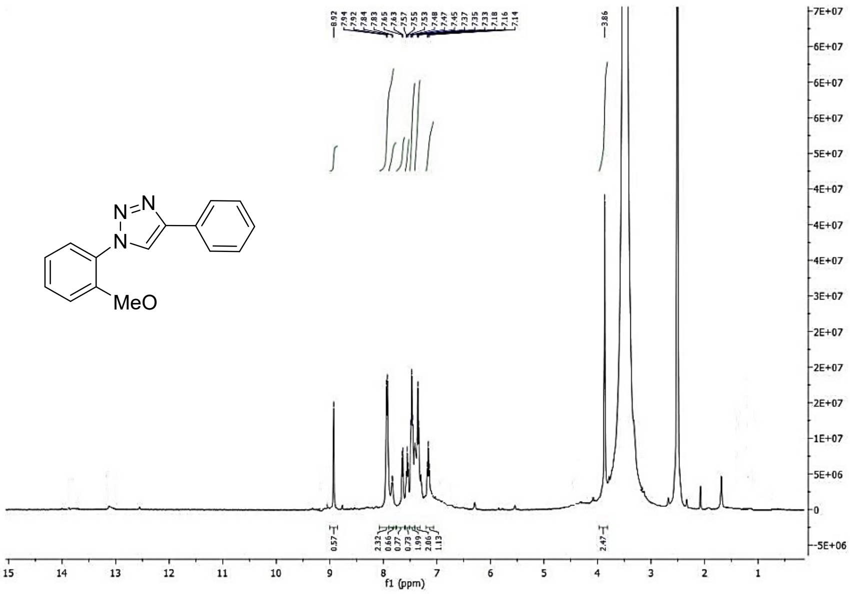

Figure S3. H NMR of 2-(4-Phenyl-1H-1,2,3-triazol-yl)methoxybenzene

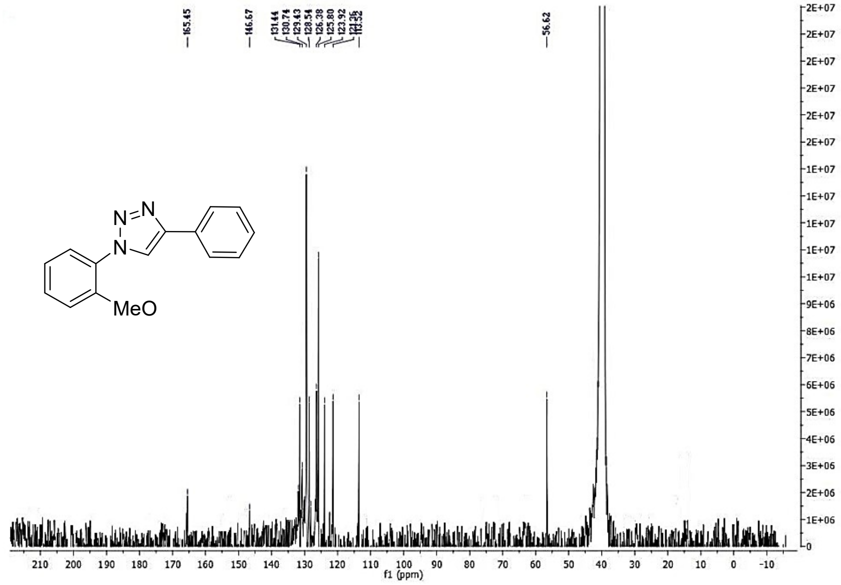

Figure S4. 13C NMR of 2-(4-Phenyl-1H-1,2,3-triazol-yl)methoxybenzene

^{1}H NMR (400 MHz, DMSO) δ 9.45 (s, 1H), 8.22 (s, 1H), 8.00 – 7.82 (m, 1H), 7.71 (d, *J* = 14.9 Hz, 2H), 7.66 – 7.56 (m, 1H), 7.53 – 7.45 (m, 1H), 7.37 (d, *J* = 5.3 Hz, 1H), 7.23 – 6.98 (m, 2H). ^{13}C NMR (101 MHz, DMSO) δ 146.45, 137.00, 135.52, 133.22, 132.45, 131.47, 130.72, 129.52,

128.63, 126.49, 125.82, 122.89, 121.64, 120.35. IR(KBr) 3465.3, 3302.4, 2136.83, 2037.23, 1591.77, 1451.61, 1248.86, 885.47 cm^{-1}.

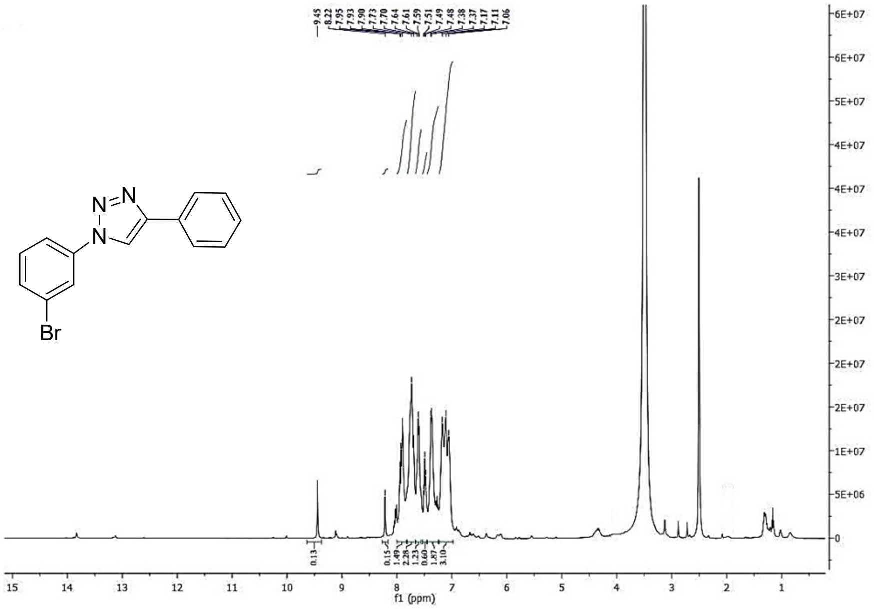

Figure S5. H NMR of 3-(4-Phenyl-1H-1,2,3-triazol-yl)bromobenzene

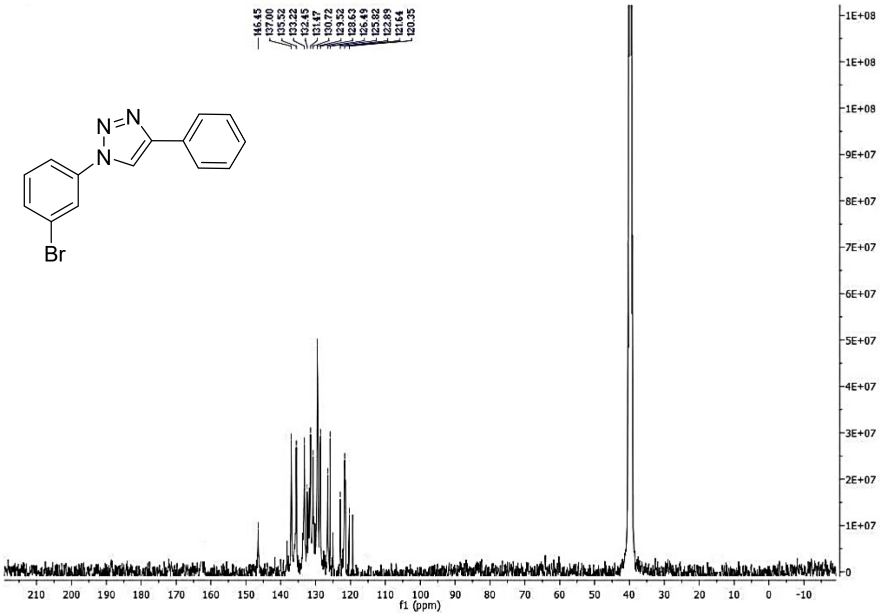

Figure S6. ^{13}C NMR of 3-(4-Phenyl-1H-1,2,3-triazol-yl)bromobenzene

^{1}H NMR (400 MHz, DMSO) δ 9.55 (s, 1H), 9.39 (s, 1H), 8.12 (d, *J* = 8.1 Hz, 1H), 7.87 (dd, *J* = 24.9, 7.4 Hz, 4H), 7.70 (d, *J* = 7.2 Hz, 2H), 7.36 – 7.19 (t, 1H), 7.07 (t, *J* = 6.8 Hz, 1H). ^{13}C NMR (101 MHz, DMSO) δ 171.79, 141.22, 140.03, 133.60, 131.04, 130.67, 129.47, 128.77, 128.41, 128.23, 127.63, 125.81, 125.24, 125.05, 119.18. IR(KBr) 3633.32, 3532.79, 3356.88, 2140.95, 2037.925, 1580.87, 1526.17, 1400.21, 1209.61 cm^{-1}.

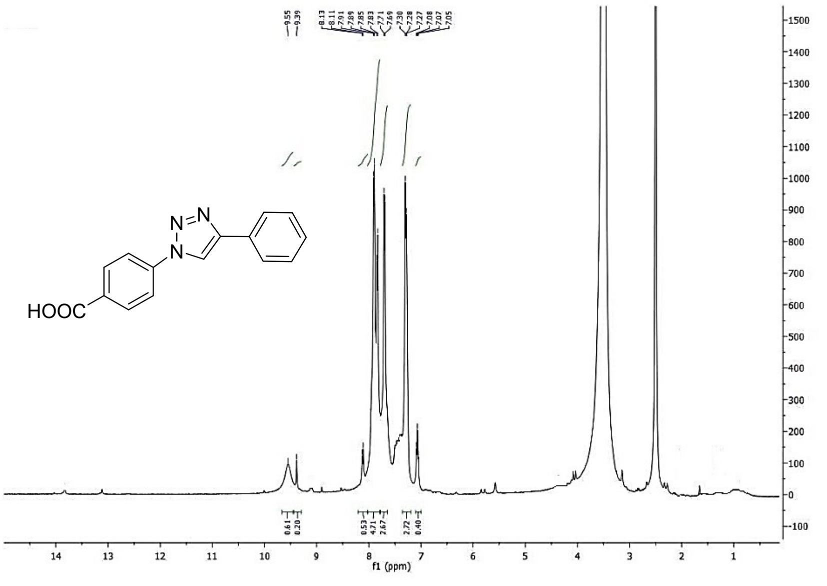

Figure S7. H NMR of 4-(4-Phenyl-1H-1,2,3-triazol-yl)benzoic acid

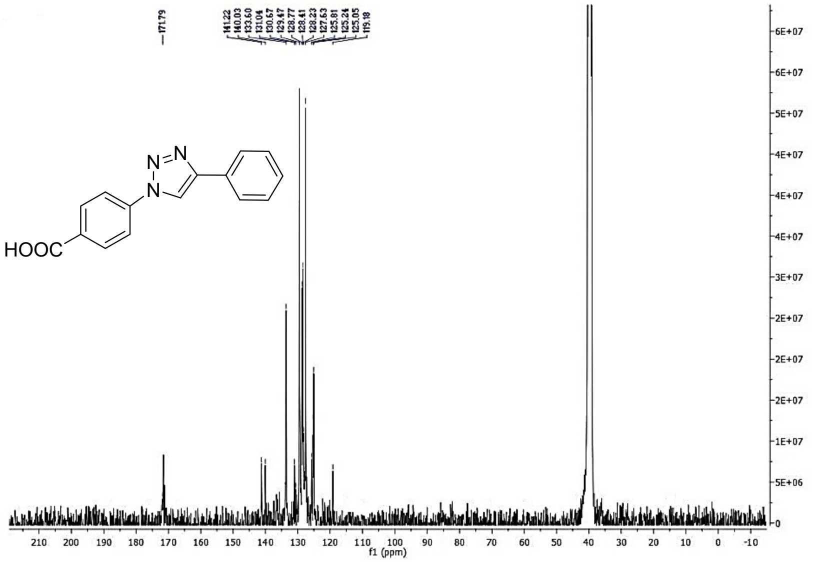

Figure S8. ^{13}C NMR of 4-(4-Phenyl-1H-1,2,3-triazol-yl)benzoic acid

^{1}H NMR (400 MHz, DMSO) δ 9.35 (s, 1H), 7.85 (t, *J* = 7.4 Hz, 2H), 7.65 (t, *J* = 7.4 Hz, 2H), 7.55 – 7.40 (m, 1H), 6.97 (t, *J* = 8.9 Hz, 2H), 6.89 – 6.75 (m, 2H). ^{13}C NMR (101 MHz, DMSO) δ 164.17, 161.63, 159.70, 139.19, 136.56, 134.18, 133.44, 129.44, 128.72, 125.81, 124.98, 113.49, 113.30, 112.42. IR(KBr) 3390.13, 3355.9, 2126.20, 2036.96, 1591.51, 1505, 1455.27, 1228.05, 1160.32, 1009.13, 903.78, 823.73 cm^{-1}.

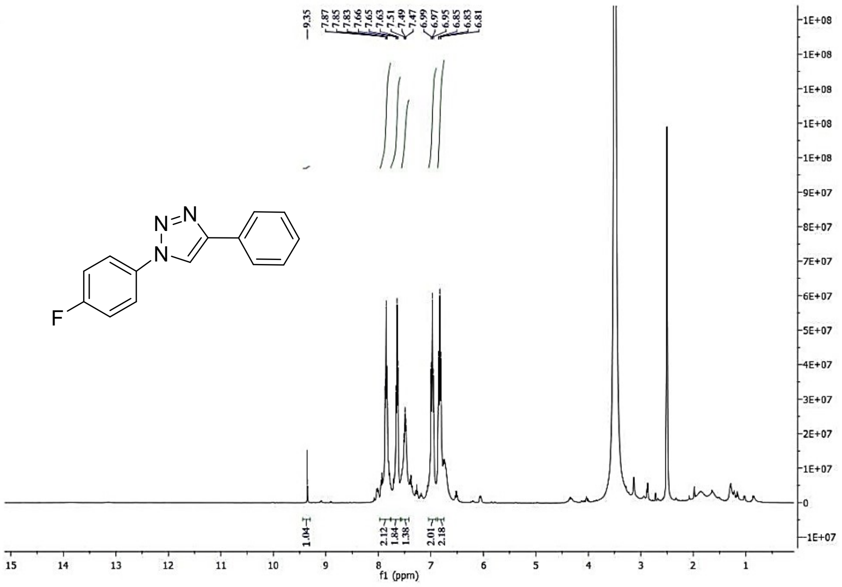

Figure S9. H NMR of 4-(4-Phenyl-1H-1,2,3-triazol-yl)fluorobenzene

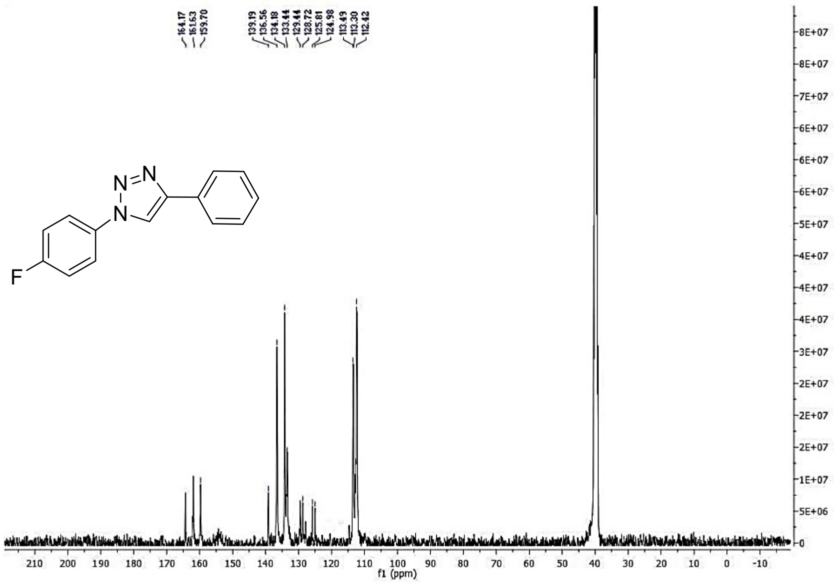

Figure S10 . ^{13}C NMR of 4-(4-Phenyl-1H-1,2,3-triazol-yl)fluorobenzene

^{1}H NMR (400 MHz, DMSO) δ 9.37 (s, 1H), 7.96 (d, *J* = 5.0 Hz, 2H), 7.74 (d, *J* = 6.9 Hz, 1H), 7.63 (t, *J* = 7.7 Hz, 1H), 7.50 (dd, *J* = 14.6, 7.3 Hz, 2H), 7.42 – 7.33 (m, 2H), 7.30 (d, *J* = 6.9 Hz, 2H). ^{13}C NMR (101 MHz, DMSO) δ 147.81, 137.06, 134.51, 131.20, 130.67, 130.43, 130.17, 129.47, 129.20, 128.73, 127.70, 125.81, 120.40, 120.14. IR(KBr) 3390.31, 3357.03, 2138.24, 2037.32, 1640.60, 1599.93, 1443.47, 1415.70, 1275.46, 1001.88 cm^{-1}.

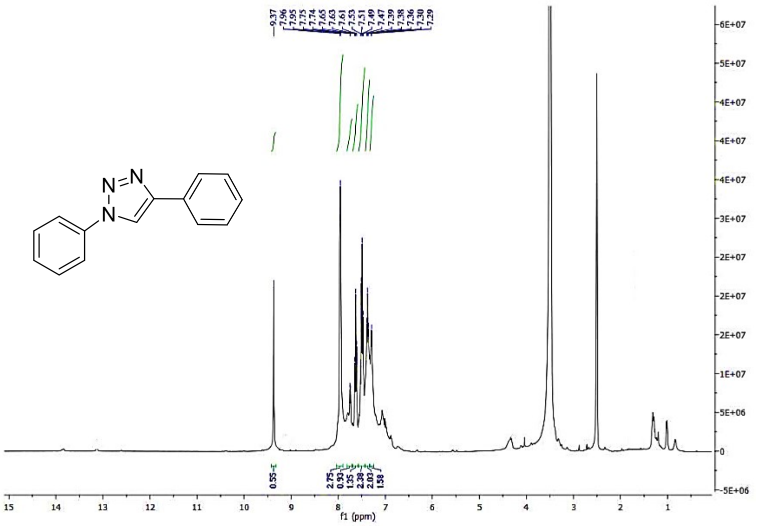

Figure S11. H NMR of (4-Phenyl-1H-1,2,3-triazol-yl) benzene

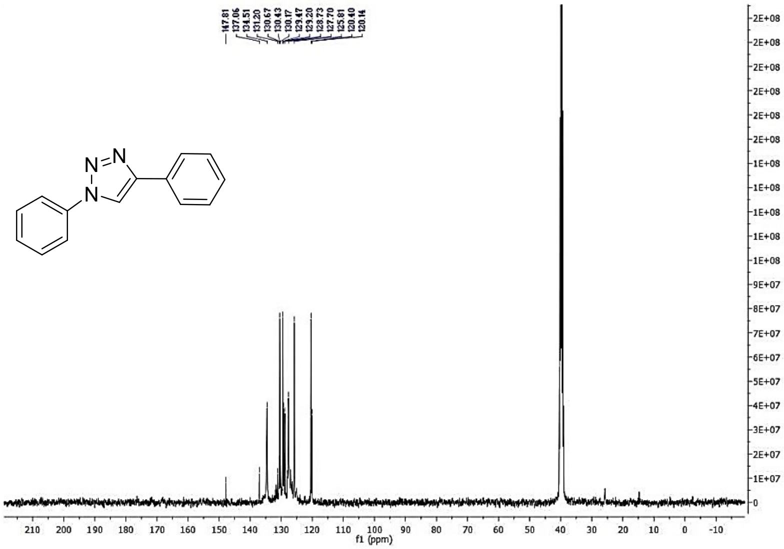

Figure S12 . ^{13}C NMR of (4-Phenyl-1H-1,2,3-triazol-yl) benzene

^{1}H NMR (400 MHz, DMSO) δ 8.94 (s, 1H), 7.79 (d, J = 2.2 Hz, 1H), 7.65 (d, J = 6.6 Hz, 1H), 7.45 – 7.34 (m, 2H), 7.33 (dd, J = 7.6, 4.3 Hz, 1H), 7.18 (d, J = 1.6 Hz, 2H), 7.09 – 6.88 (m, 1H), 3.79 (s, 3H). ^{13}C NMR (101 MHz, DMSO) δ 171.74, 138.69, 138.34, 137.51, 134.52, 130.80, 128.68, 128.49, 127.52, 127.22, 125.85, 124.94, 123.10, 114.89, 55.64. IR(KBr) 3390.36, 3356.94, 2128.95, 2037.14, 1440.32, 1598.71, 1254.76, 1003.71, 639.36 cm^{-1}.

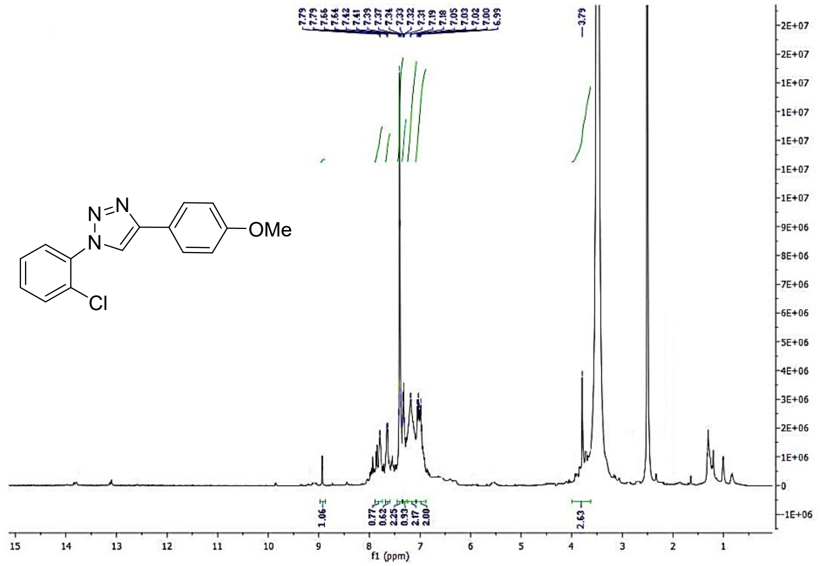

Figure S13. H NMR of 2(4[4-methoxyPhenyl] 1H-1,2,3-triazol-yl) chlorobenzene

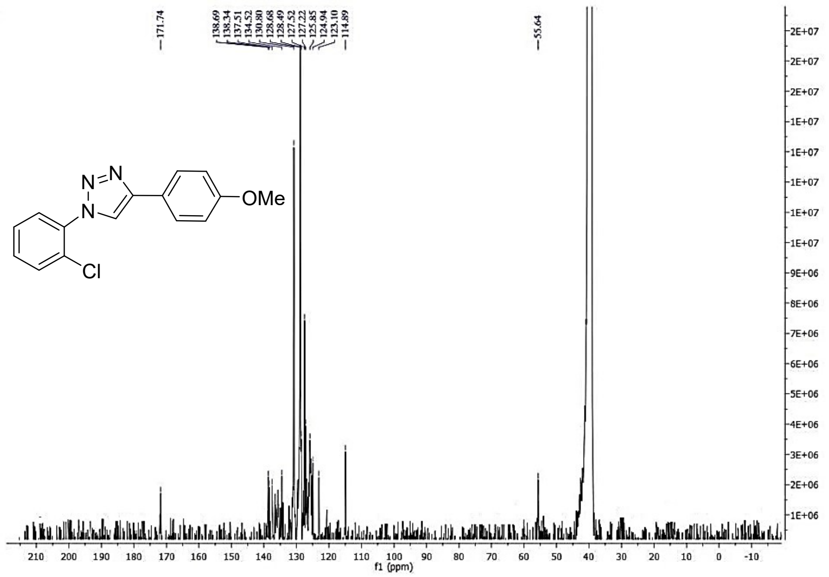

Figure S14. ^{13}C NMR of 2(4[4-methoxyPhenyl] 1H-1,2,3-triazol-yl) chlorobenzene

^{1}H NMR (250 MHz, DMSO) δ 9.05 (s, 1H), 7.91 (d, *J* = 7.0 Hz, 1H), 7.73 (s, 1H), 7.54 (d, *J* = 6.1 Hz, 1H), 7.48 – 7.31 (m, 2H), 7.21 (s, 2H), 7.06 (d, *J* = 4.6 Hz, 1H), 6.95 (d, *J* = 6.1 Hz, 1H). ^{13}C NMR (63 MHz, DMSO) δ 145.65, 133.87, 132.35, 131.06, 129.45, 128.86, 128.35, 125.77, 123.82, 120.73, 120.35, 119.96, 118.81, 108.83. IR(KBr) 3390.10, 3356.84, 2145.79, 2037.37, 1593.69, 1455.98, 1392.92, 639.11 cm^{-1}.

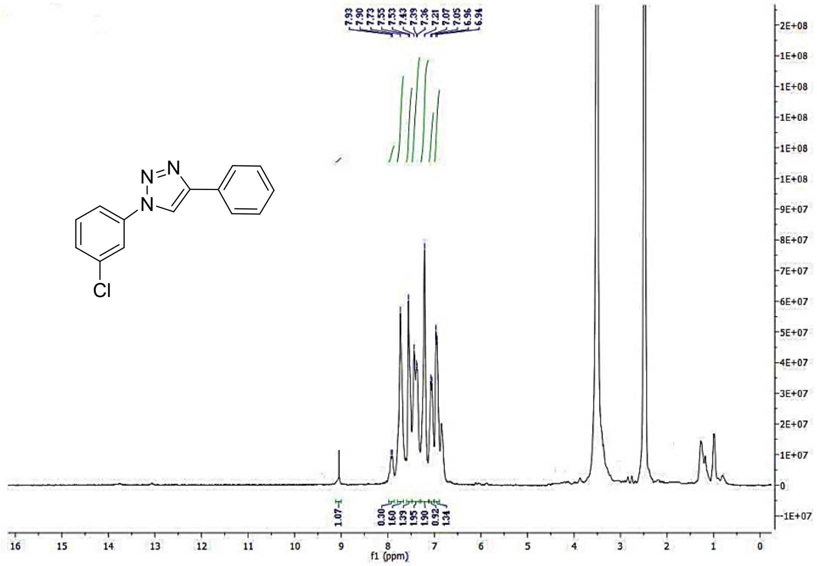

Figure S15. H NMR of 3-(4-Phenyl-1H-1,2,3-triazol-yl)chlorobenzene

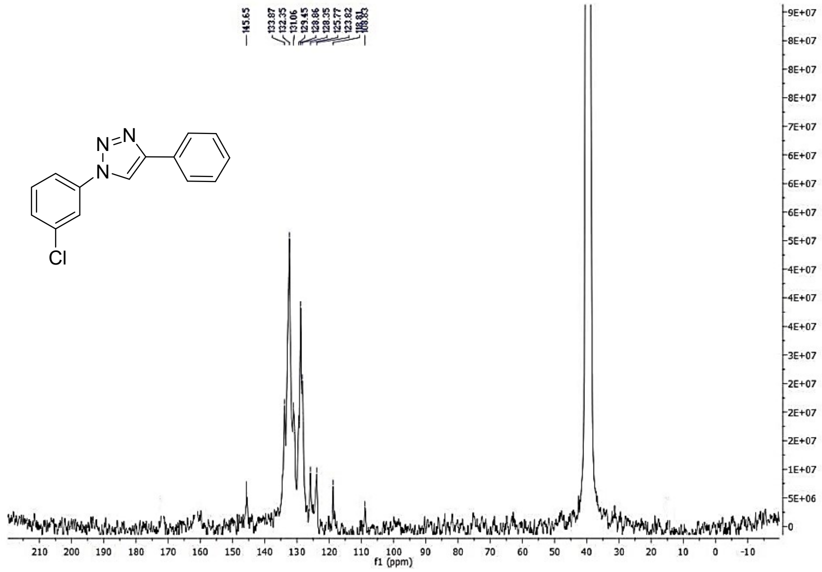

Figure S16. ^{13}C NMR of 3-(4-Phenyl-1H-1,2,3-triazol-yl)chlorobenzene

^{1}H NMR (400 MHz, DMSO) δ 9.29 (s, 1H), 7.96 (dd, *J* = 29.8, 8.1 Hz, 3H), 7.78 (d, *J* = 7.7 Hz, 1H), 7.55 (t, *J* = 7.3 Hz, 1H), 7.50 – 7.36 (m, 1H), 7.23 (d, *J* = 8.5 Hz, 2H), 6.93 (d, *J* = 8.2 Hz, 1H), 3.90 (s, 3H). ^{13}C NMR (101 MHz, DMSO) δ 164.09, 159.82, 158.00, 151.81, 151.12, 136.33, 130.89, 130.58, 129.50, 128.68, 125.85, 122.16, 120.17, 115.46, 56.12. IR(KBr) 3389, 3357.10, 2143.89, 2037.22, 1519.32, 1463.96, 1345.84, 1248.53, 1129.04, 1004.36 cm^{-1}.

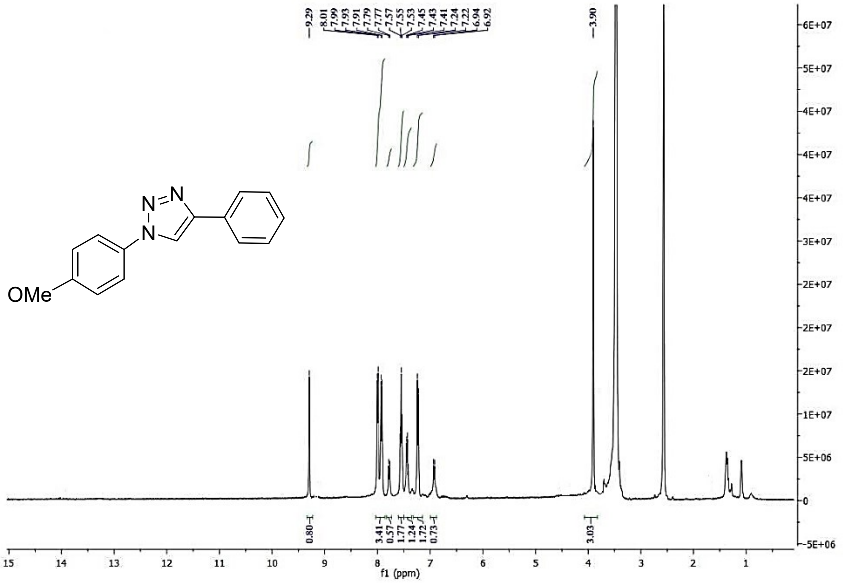

Figure S17. H NMR of 4-(4-Phenyl-1H-1,2,3-triazol-yl)anisole

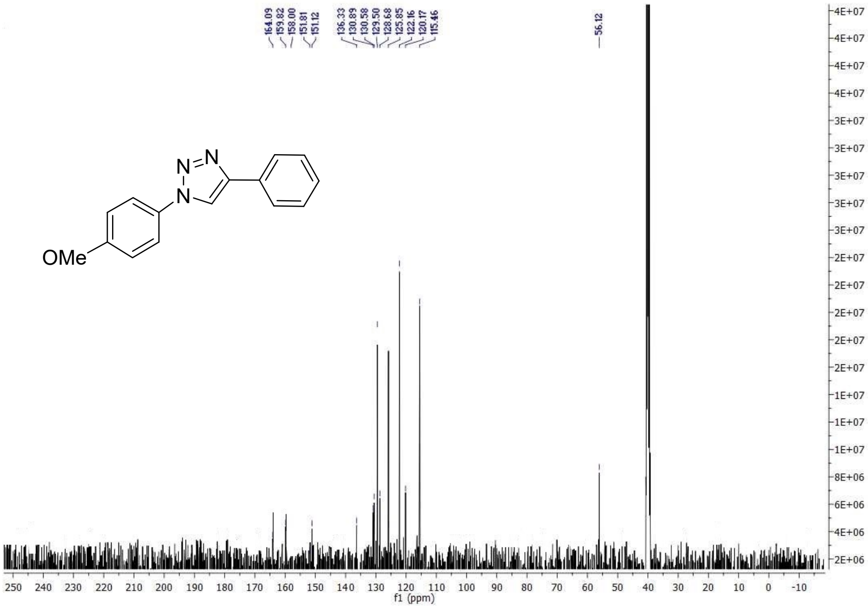

Figure S18. ^{13}C NMR of 4-(4-Phenyl-1H-1,2,3-triazol-yl)anisole

^{1}H NMR (400 MHz, DMSO) δ 9.10 (s, 1H), 8.01 – 7.83 (m, 1H), 7.44 (dd, *J* = 29.0, 7.9 Hz, 1H), 7.12 (t, *J* = 7.6 Hz, 3H), 6.73 (d, *J* = 7.9 Hz, 3H), 6.62 – 6.48 (m, 1H), 3.14 (s, 1H). ^{13}C NMR (101 MHz, DMSO) δ 157.99, 147.76, 138.18, 130.68, 130.40, 129.76, 129.44, 125.72, 121.57, 119.01, 118.86, 117.03, 116.08, 115.73, 64.12. IR(KBr) 3390.61, 3358.4, 2161.24, 2037.29, 1656.65, 1489.73, 1345.57, 1259.71, 1130.55, 1000.90 cm^{-1}.

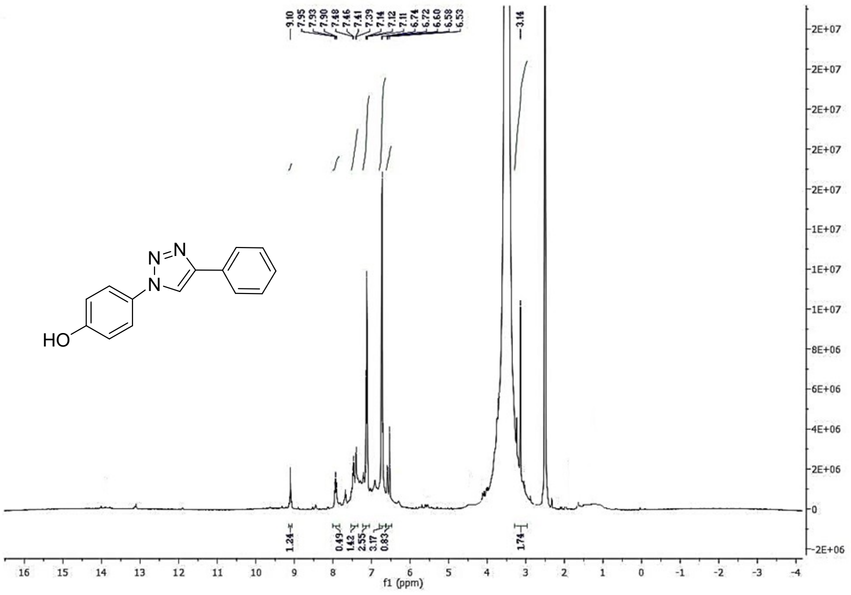

Figure S19. H NMR of 4-(4-Phenyl-1H-1,2,3-triazol-yl)phenol

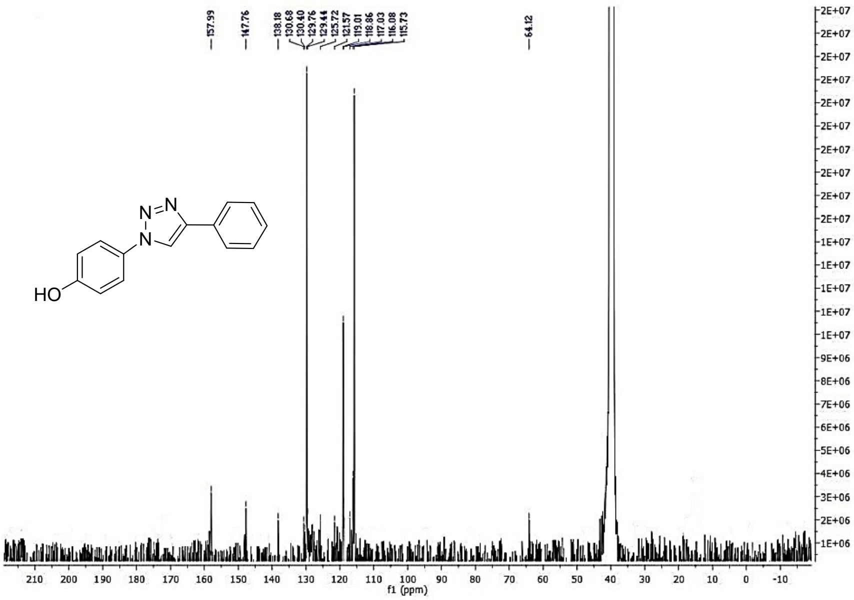

Figure S20. ^{13}C NMR of 4-(4-Phenyl-1H-1,2,3-triazol-yl)phenol
